# Incidence and Mortality of Dementia-Related Missing and Their Associated Factors: An Ecological Study in Japan

**DOI:** 10.2188/jea.JE20200113

**Published:** 2021-06-05

**Authors:** Shunsuke Murata, Misa Takegami, Daisuke Onozuka, Yuriko Nakaoku, Akihito Hagihara, Kunihiro Nishimura

**Affiliations:** Department of Preventive Medicine and Epidemiology, National Cerebral and Cardiovascular Center Research Institute, Osaka, Japan

**Keywords:** dementia-related missing incident, death after dementia-related missing, dementia, ecological study

## Abstract

**Background:**

Dementia-related missing and subsequent deaths are becoming serious problems with increases in people with dementia. However, there are no sufficient studies investigating the incidence rate, the mortality rate, and their risk factors.

**Methods:**

An ecological study aggregated at the Japanese prefectural level was conducted. Dementia-related missing persons cases and deaths in 2018 were extracted from the statistics of the National Police Agency in Japan. We extracted variables about older adults’ characteristics, care, and safety as candidate variables considered to be relevant to dementia-related missing persons cases and deaths. Associations of the candidate variables with the incidence and mortality rates were analyzed using the generalized linear model (family: quasi-poisson, link: log) adjusted for confounding factors (proportion of older adults and gross prefectural product).

**Results:**

The incidence rate and mortality rate per 100,000 person-year was 21.72 and 0.652 in Japan, respectively. One facility increase in the number of nursing care facilities for older adults per 100,000 persons aged 65-years-old or more was associated with a 7.9% (95% confidence interval [CI], 3.3–12.4%) decrease in the incidence rate. One increase in the number of public health nurses per 100,000 persons was associated with a 3.2% (95% CI, 1.6–4.9%) decrease in the incidence rate. A ten percent increase in the proportion of people who live in an urban area was associated with a 20.3% (95% CI, 8.7–33.2%) increase in the incidence rate and a 12.9% (95% CI, 5.6–19.8%) decrease in the mortality rate.

**Conclusions:**

Identified associated factors may be useful for managing or predicting dementia-related missing persons cases and associated deaths.

## INTRODUCTION

The prevalence of Alzheimer’s disease has increased rapidly with the growth of the older adult population, and it is expected that the 33.9 million cases currently estimated worldwide will triple by 2050.^1^^,^^2^ Wandering behavior is one of the most troublesome dementia behavior disturbances and leads to several adverse outcomes such as fractures, getting lost, and untimely death.^3^^–^^5^ The number of dementia-related missing persons cases is increasing, with 16,927 cases per year being reported in Japan.^6^ Moreover, wandering behavior is associated with a high care burden for families^7^; they are sometimes sued after accidents that are dementia-related missing persons cases.^8^ There are no efficacious ways to prevent people with dementia from getting lost.^9^ Therefore, there is a need to identify the risk factors for dementia-related missing incidents and the subsequent death and to develop an efficacious way to manage them.

Some studies investigated the characteristics of missing persons cases and their risk factors. One study found that 75% of older adults with dementia died after going missing due to hypothermia or drowning and that the proportion of older adults living alone who died after a missing event was higher than those found alive.^10^ Another study reported that persons who died after going missing were more likely to get lost at night (from 18:00 to 5:59) and that it took a longer time to submit a missing person’s report for persons who died than for persons who were found alive.^11^ Another retrospective observational study found that a higher outdoor landmark density was a significant geospatial factor for missing incidents related to dementia.^12^

There are several studies that investigated the risk factors for missing incidents in persons with dementia and the differences between death cases after dementia-related missing and alive cases after dementia-related missing. However, there is no study that investigates the risk factors for deaths after a dementia-related missing event. Also, few studies have investigated the environmental risk factor for dementia-related missing incidents, such as the care and safety status of older adults. Additionally, there are no data about the incidence rate and mortality rate of dementia-related missing events, despite these being crucial information in determining how often missing events and subsequent deaths occur.

Therefore, the purpose of this study was to describe the incidence rate and mortality rate of dementia-related missing events, and to investigate the risk factors for missing incidents and subsequent deaths.

## METHODS

### Design and data source

An exploratory ecological design was used by aggregating data by prefecture; Japan has 47 prefectures. The National Police Agency gave our group the data about dementia-related missing incidents and the subsequent deaths. Other data were extracted from the Japanese government statistics open data (e-stat).^13^ Table 1 shows a detailed explanation of the variables and data source of this study. This study did not require an institutional review board approval, since only aggregate data were used in this study.

**Table 1.** Detailed summary of variables in this study

Variable name	Unit	Type	Calculation methods	Data source	Year
Dementia-related missing event	Case	Outcome	—	Status of missing from The National Police Agency	2018
Death after dementia-related missing	Case	Outcome	—	Status of missing from The National Police Agency	2018
The number of nursing care facilities for older adults per 100,000 persons aged 65 years old or more	Facility per 100,000 Persons aged 65 years old or more	Explanatory variables	The number of nursing care facilities for older adults/the number of persons aged 65 years old or more	Survey of Institutions and Establishments for Long-term Care and Population Census or estimated population	2017
The number of policemen per 1,000 persons	Person per 1,000 persons	Explanatory variables	The number of policemen/total population	Local government capacity management survey and Population Census or estimated population	2017
Welfare costs for older adults per one person aged 65 years old or more	Thousand yen per person aged 65 years old or more	Explanatory variables	Welfare costs for older adults/the number of persons aged 65 years old or more	Local financial status survey and Population Census or estimated population	2016
The number of public health nurses per 100,000 persons	Person per 100,000 persons	Explanatory variables	The number of public health nurse/total population	Report on Public Health Administration and Services and Population Census or estimated population	2016
The number of local welfare officers per 100,000 persons	Person per 100,000 persons	Explanatory variables	The number of local welfare officer per 100,000 persons	Report on Social Welfare Administration and Services and Population Census or estimated population	2016
Late elderly medical expenses per one insured person	Thousand yen per insured person	Explanatory variables	Late elderly medical expenses/ insured person	Latter-year medical care business annual report and Population Census or estimated population	2016
The proportion of people who live in urban areas	Percent	Explanatory variables	The number of people who live in urban areas/total population	Population Census	2015
The proportion of households with older adults couples only per household	Percent	Explanatory variables	The number of households with older adults couples only per household/total households	Population Census	2015
The proportion of households with older adults living alone per household	Percent	Explanatory variables	The number of households with older adults living alone per household/total households	Population Census	2015
Population aged 40 years old or more	Persons	Offset	—	Estimated population	2018
Proportion of older adults	Percent	Confounding variable	The number of persons aged 65 years old or more/total population	Estimated population	2018
Gross prefectural product	Million yen	Confounding variable	—	Prefectural Economic Calculation	2015

—, Not applicable.

### Measurements

#### Dementia-related missing events and deaths after dementia-related missing

Missing person events and related deaths in 2018 were used as outcomes in present study.^6^ In Japan, there are rules regarding how to find missing persons based on related laws.^14^ This states that a missing person is classified as such when a missing person’s report has been submitted by family or other related persons to the police. Missing is defined as having left the living place and the person’s location is unknown. Death after submitting a missing person’s report is treated as a death after dementia-related missing event. There is the possibility that an individual with dementia gets lost repeatedly and multiple missing persons reports are submitted. If the family or other related persons report that a missing person has dementia, this case is treated as dementia-related missing incident. Only in dementia-related missing events, more detail aggregated data by age (40–49, 50–59, 60–69, 70–79, and 80 years or older), sex, and prefectures can be collected.

#### Total population

In order to calculate the incidence rate, death rate, and age-standardized incidence rate, we obtained total population data from annual reports which are based on the population Residential Register of Japan.^13^ We extrapolated data from those who were aged 40 years or older, then examined data from 2018 by age groups (40–49, 50–59, 60–69, 70–79, and 80 years or older).

#### Candidate explanatory variables

We selected the candidate explanatory variables based on environmental and economic indicators that are expected to be associated with dementia-related missing events and related deaths as the older adults’ characteristics, care, and safety. The following variables were selected: the number of nursing care facilities for older adults per 100,000 persons aged 65 years or older, the number of policemen per 1,000 persons, welfare costs for older adults per one person aged 65 years or older, the number of public health nurses per 100,000 persons, the number of local welfare officers per 100,000 persons, late elderly medical expenses per one insured person, the proportion of people who live in the urban area, the proportion of households with only one older adult couple per household, and the proportion of households with older adults living alone per household.

#### Confounding variables

We extracted the data of the proportion of older adults and the gross prefectural product. The proportion of older adults and gross prefectural product were used as confounding variables.

### Statistical analysis

The incidence rate and mortality rate of dementia-related missing persons in the Japanese population and each prefecture were calculated by dividing dementia-related missing cases and the cases of death by the total population. Detailed missing incidence information based on age (40–49, 50–59, 60–69, 70–79, and 80 years or older) and sex were extracted in the case of missing events due to dementia. Therefore, the age-standardized incidence rate (direct methods) and age-sex specific incidence rate were calculated only for missing events due to dementia. The age-standardized incidence rate was calculated using the “epitools” package.^15^ Additionally, the generalized linear model (family: quasi-poisson, link: log) was applied to investigate the missing incidences among prefectures, age category, and sex, since there was overdispersion in the missing cases.^16^ The dependent variables were missing event and prefecture, and the age category (40–49, 50–59, 60–69, 70–79, and 80 years or older), sex, and prefecture were entered as the independent variables. The total population of each category was included as an offset variable.

Generalized linear models (family: quasi-poisson, link: log) were also applied to explore correlated factors for dementia-related missing incidents or subsequent deaths, since there was overdispersion in both.^16^ The total population aged at least 40 years was included as an offset variable. Candidate explanatory variables with *P*-values <0.05 were adjusted for the proportion of older adults and gross prefectural product. The incidence rate ratios (IRRs) or the mortality rate ratios with 95% confidence intervals (CIs) were calculated. As a sensitivity analysis, the same analyses of missing incidents were conducted only in older adults (65 years or older).

Statistical significance was set at *P* < 0.05, and all analyses were conducted using R (version 3.6.1; R Foundation for Statistical Computing, Vienna, Austria).

## RESULTS

### Incidence rate and mortality rate of dementia-related missing and demographic data

**Figure 1.**
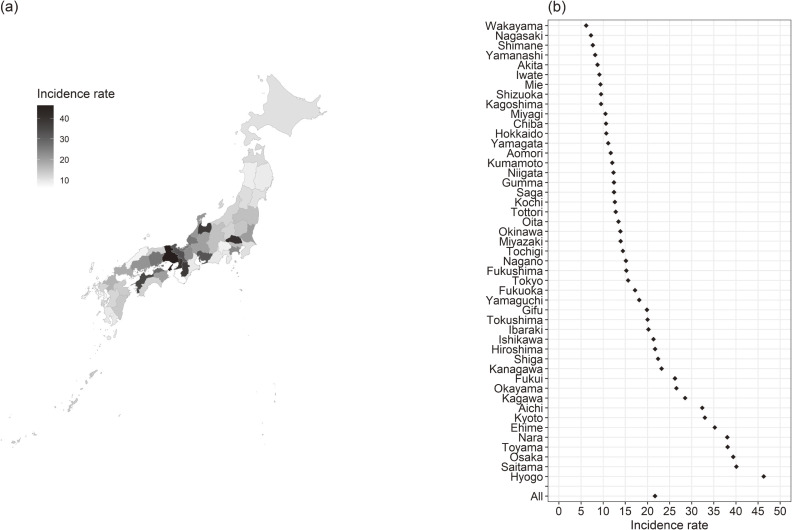
Incidence rate of missing persons with dementia (per 100,000 person-years). Map of Japan showing the incidence rate of missing persons with dementia (per 100,000 person-years) (a), the incidence rate (per 100,000 person-years) in Japan and in each prefecture (b).

**Figure 2.**
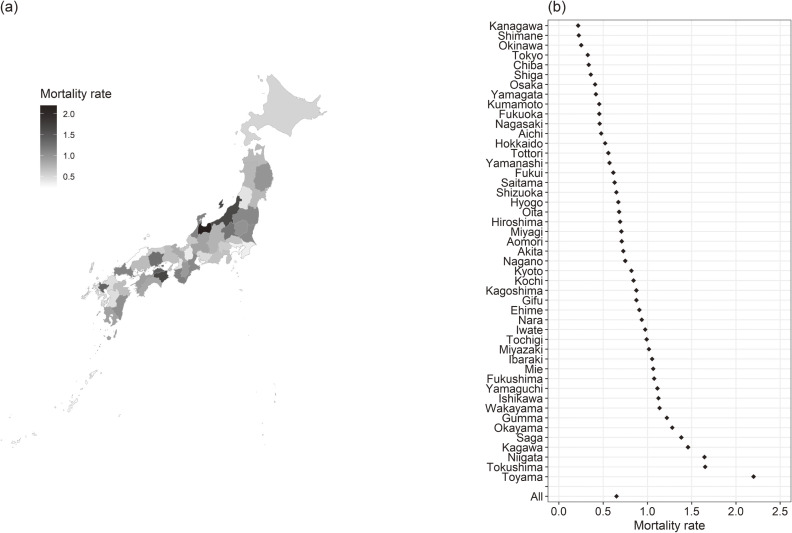
Mortality rate of missing persons with dementia (per 100,000 person-years). Map of Japan showing the mortality rate of missing in persons with dementia (per 100,000 person-years) (a), and the mortality rate (per 100,000 person-years) in Japan and in each prefecture (b).

The total incidence of dementia-related missing persons cases and the total subsequent deaths in Japan were 16,927 cases and 508 cases, respectively. The incidence rate and mortality rate per 100,000 person-year in Japan was 21.72 and 0.652, respectively. The prefectural-level range of dementia-related missing cases was from 34 to 2,117. The prefectural-level range of deaths after dementia-related missing incidents was from 1 to 28. Figure 1 and Figure 2 depict the incidence rate and mortality rate in Japan and in each prefecture.

Table 2 shows dementia-related missing events, subsequent death, total population, and candidate explanatory variables. The mean proportion of older adults was 30.07% (standard deviation [SD], 2.97%). The mean proportion of households with older adults living alone was 11.36% (SD, 1.86%).

**Table 2.** Description of dementia-related missing event, subsequent death, total population, and candidate explanatory variables

Variable	Overall (*n* = 47)
The number of discovered cases after dementia-related missing event	345.26 (488.91)
Number of dementia-related missing events	360.15 (496.34)
Number of dementia-related missing events in older adults	349.94 (482.01)
Incidence rate of dementia-related missing [per 100,000 person-year]	18.62 (10.38)
The number of deaths after dementia-related missing	10.81 (6.76)
Mortality rate of dementia-related missing [per 100,000 person-year]	0.82 (0.42)
Population of older adults	756,914.89 (681,602.10)
Population of people aged 40 years old or more	1,658,510.64 (1,630,361.63)
Proportion of older adults [%]	30.07 (2.97)
Gross prefectural Product [million yen]	11,628,733.85 (16,58,308.01)
The number of nursing care facilities for the older adults [building per 100,000 older adults]	23.25 (4.59)
The number of policemen [person per 1,000 persons]	1.96 (0.30)
Welfare costs for older adults [thousand yen per older adult aged 65 years old or more]	207.71 (22.76)
The number of public health nurses [per 100,000 persons]	49.55 (12.68)
The number of local welfare officers [per 100,000 persons]	225.90 (55.41)
Late elderly medical expenses [thousand yen per insured]	930.35 (106.56)
The proportion of people who live in urban areas [%]	52.53 (18.92)
The proportion of households with older adults staying as a couples only [%]	12.09 (1.57)
The proportion of households with older adults living alone [%]	11.36 (1.86)

All data was shown as mean (standard deviation).

### Age- and sex-specific incidence rate of missing persons and age-standardized incidence rate

The age-sex specific incidence rates (per 100,000 person-years) of 40–49 year old men, 50–59 year old men, 60–69 year old men, 70–79 year old men, men aged ≥80 years, 40–49 year old women, 50–59 year old women, 60–69 year old women, 70–79 year old women, and women aged ≥80 years were 0.07, 0.80, 8.54, 49.22, 128.03, 0.02, 0.84, 7.44, 38.40, and 53.51, respectively (eFigure 1a). The age-standardized incidence rate ranged from 3.4 to 28.5 (eFigure 1b). eFigure 1c depicts the IRR from the generalized linear model that investigated the difference from the missing incidence by prefecture, age, and sex. Our analysis showed significant differences in prefectures, age, and sex (range of IRRs: 0.32 to 2.62). Older age was associated with a higher incidence rate of dementia-related missing events. Men were more likely to get lost compared to women (IRR 1.74; 95% CI, 1.66–1.83). eTable 1 shows the sex and age distributions of the total dementia-related missing events. Dementia-related missing events of men and women were 5,065 and 3,792 cases, respectively.

### Associated factors for missing incidents in persons with dementia

**Figure 3.**
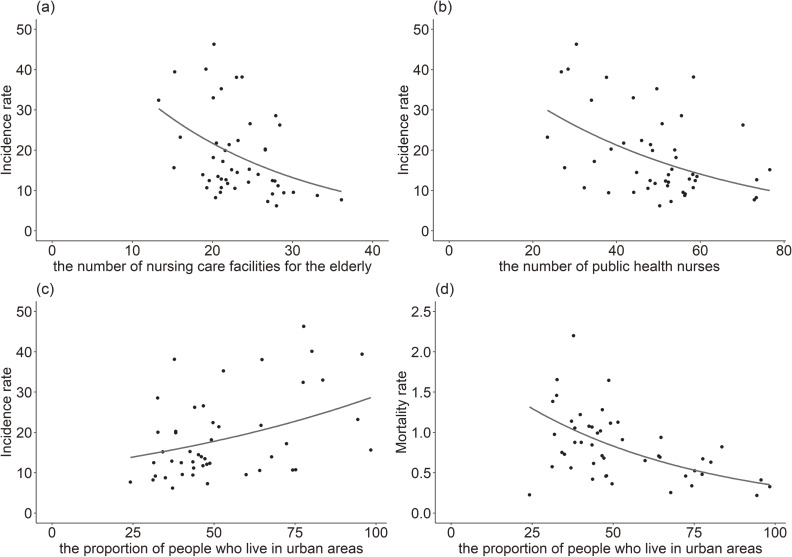
Scatter plot and estimated value from generalized linear model. Points suggest each value and lines suggest estimated value from generalized linear model. (a) is about the incidence rate and the number of nursing care facilities for the elderly, (b) is about the incidence rate and the number of public health nurses, (c) is about the incidence rate and the proportion of people who live in urban areas, and (d) is about the mortality rate and the proportion of people who live in urban areas.

Table 3 shows the results of the associated factors in dementia-related missing incidents. We found that the number of nursing care facilities for older adults, number of public health nurses, and proportion of people who live in the urban area were associated with dementia-related missing incidents. An increase in the number of nursing care facilities for older adults per 100,000 persons aged 65 years or older by one was associated with a 7.9% (95% CI, 3.3–12.4%) decrease in missing incidents. An increase in the number of public health nurse per 100,000 persons by one was associated with a 3.2% (95% CI, 1.6–4.9%) decrease in missing incidents. A ten percent increase in the proportion of people who live in the urban area was associated with a 20.3% (95% CI, 8.7–33.2%) increase in missing incidents. Figure 3a, Figure 3b, and Figure 3c depicts scatter plots and an estimated incidence rate from the non-adjusted generalized linear models results of the number of nursing care facilities for older adults, number of public health nurses, and proportion of people who live in the urban area to show the estimated incidence rate with the distribution of each value. eFigure 2a, eFigure 2b, and eFigure 2c depicts scatter plots with the prefecture and the number of nursing care facilities for older adults, number of public health nurses, and proportion of people who live in the urban area to determine the specific value of each prefecture. For example, the number of nursing care facilities for the elderly, number of public health nurses, and proportion of people who live in the urban area were 15.2, 27.6, and 98.4, respectively, in Tokyo, which is the capital of Japan. A sensitivity analysis also showed that a higher number of public health nurses and nursing care facilities, and a lower proportion of people who live in the urban area were associated with a lower incidence rate of dementia-related missing events (eTable 2).

**Table 3.** Association of the candidate variables with missing incident and death after missing in persons with dementia: generalized linear model

	Missing incident				Death after missing			
	
	Crude model		Adjusted model^a^		Crude model		Adjusted model^a^	
	
	IRR (95% CI)	*P*	IRR (95% CI)	*P*	IRR (95% CI)	*P*	IRR (95% CI)	*P*
The number of nursing care facilities for older adults	0.951 (0.921–0.982)	0.004	0.921 (0.876–0.967)	0.002	1.071 (1.045–1.097)	<0.001	1.033 (0.997–1.071)	0.081
The number of policemen	0.962 (0.674–1.336)	0.824	—		0.660 (0.441–0.953)	0.040	0.976 (0.573–1.628)	0.927
Welfare costs for older adults	0.994 (0.988–1.000)	0.055	—		1.004 (0.998–1.011)	0.218	—	
The number of public health nurses	0.980 (0.969–0.990)	0.001	0.968 (0.951–0.984)	<0.001	1.020 (1.011–1.030)	<0.001	1.002 (0.989–1.016)	0.723
The number of local welfare officers	0.998 (0.995–1.000)	0.091	—		1.005 (1.003–1.007)	<0.001	0.999 (0.994–1.005)	0.761
Late elderly medical expenses	1.001 (1.000–1.002)	0.140	—		0.999 (0.998–1.001)	0.275	—	
The proportion of people who live in urban areas	1.010 (1.003–1.017)	0.005	1.019 (1.008–1.029)	0.001	0.982 (0.978–0.987)	<0.001	0.986 (0.978–0.994)	0.002
The proportion of households with older adults living as a couples	1.023 (0.932–1.126)	0.642	—		1.172 (1.068–1.289)	0.002	0.973 (0.869–1.091)	0.640
The proportion of households with older adults living alone	1.006 (0.914–1.104)	0.899	—		1.016 (0.919–1.120)	0.751	—	

CI, confidence interval; IRR, incidence rate ratio; —, Not applicable.

^a^Adjusted models were applied using explanatory variables which are statistically significant in crude model with proportion of older adults and gross prefectural product as confounding factor.

### Associated factors for deaths after dementia-related missing person events

Table 3 also shows the results of the associated factors for deaths after dementia-related missing person events. Our results showed that the proportion of people who live in urban areas was associated with deaths after dementia-related missing events. A ten percent increase in the proportion of people who live in urban areas was associated with a 12.9% (95% CI, 5.6–19.8%) decrease in the mortality rate, even after adjustment for the confounding factors. Figure 3d depicts scatter plots and the estimated mortality rate from non-adjusted generalized linear models of the proportion of people who live in urban areas to show the estimated mortality rate with distribution of each value. eFigure 2d also depicts scatter plots with prefectural name of the proportion of people who live in urban areas to determine the specific value of each prefectures. For example, the mortality rates were relatively low in Tokyo, Aichi, and Osaka, which are urban prefectures in Japan.

## DISCUSSION

This is the first study to report the incidence rate and mortality rate of dementia-related missing events. Results indicated that there was a difference in the incidence rate by prefecture. Men were more likely to have missing events compared to women, and older age was associated with higher missing incidence rates. A high number of nursing care facilities for older adults, a high number of public health nurses, and a low proportion of people who live in urban areas were associated with a low rate of missing incidents. A high proportion of people who live in the urban areas was associated with a low mortality rate after missing incidents.

Our study clarified differences in the incidence rate of missing events among prefectures and found that sex (ie, men) and advanced age were associated with more missing events. Previous studies showed no significant difference in age and sex between dementia patients with missing incidence and dementia patients without missing incidence.^17^ Our study had a large sample size and strong statistical power. Our study also clarified that there were large differences in incidence rates among prefectures. Thus, our study provides further insights on characteristics of persons with dementia who get lost and additional findings about differences in prefectures in terms of the incidence rate of missing events.

Our results showed that a high proportion of people who live in urban areas was associated with a high incidence rate of dementia-related missing events. A previous retrospective observational study showed that higher outdoor landmark density is a significant geospatial factor for missing incidents in dementia,^12^ and this group suggested it is unclear why this result was found. Outdoor landmarks consist of food shops, churches, parks, hospitals, stations, traffic signals, streetlamps, towers, and so on. One possible explanation of our result may be the same as that of this retrospective observational study, since higher outdoor landmark density is thought to correspond to urban areas. People with dementia living in urban areas can go out easily. On the contrary, people with dementia living in rural areas have difficult to go out for walks, since it is frequently very far from their house to other place. People usually transport using cars or motorcycles for transportation in rural areas. Most people with dementia who get lost and die after going missing had gone out walking.^18^^,^^19^ Therefore, the difference in the ease of going out between urban and rural areas may account for the difference in the incidence rate. Additionally, our results showed that the high proportion of people who live in urban areas was associated with the low mortality rate of missing events. The possible explanation of this result might be the difference in other persons’ ease of finding the person getting lost. In urban areas, given the high population density, people who get lost are frequently seen by others. On the contrary, people who get lost in rural areas often go unseen. Therefore, our findings are partly in line with those of previous studies, expand on previous studies, and provide further insight.

This is the first study showing associations between a high number of nursing care facilities and public health nurses is associated with a low incidence rate of dementia-related missing persons, which hampers comparisons with other studies. In Japanese nursing care homes, few missing events occurred under enough management of care staff. Thus, the association between the number of nursing care facilities for older adults and incidence rate of dementia-related missing is plausible. Additionally, public health nurses visit most community-dwelling people and provide various interventions to promote healthy life. This association is also possible.

Previous studies showed that the proportion of living alone was higher in older adults with dementia who died after missing events than in older adults with dementia who were alive after missing events.^10^ However, our results could not clarify the association between proportion of older adults living alone and death after dementia-related missing events. There were differences in included participants and study designs among the studies. Our study included people aged 40 years or more, whereas previous studies included persons with dementia who get lost. This difference may lead to different results, and further research should be conducted using a large individual-level cohort study.

Our results have some practical implications. First, public health nurse activity may be efficacious to prevent dementia-related missing events. Further research should be conducted to clarify the efficacy of this management method using an intervention study. Second, the proportion of people who live in urban areas and the number of nursing care facilities for older adults can help prefectures or cities to predict missing incidence rates and the mortality rate of missing persons. Different strategies should be considered according to prefecture and urban or rural living situation.

Despite the important findings of this study, its limitations should be taken into account. First, the present study design was an ecological study. Therefore, ecological fallacy exists. Further research should be conducted by collecting individual-level data and using case control study that is feasible. However, cases of death after going missing and dementia-related missing cases are rare. Therefore, sampling should be conducted at the country-level. Second, we could not assess cases that were not reported to the police despite the occurrence of missing event. These unreported missing cases are possible when the persons live alone, have no close neighbors, and receive no long-term care insurance services. Though these situations may be rare, dementia-related missing event may also be underestimated. Third, our study had no information about cases that were not found long after the person had gone missing. Though most of these cases are expected to be dead, this study did not include those cases. Thus, death after dementia-related missing events may be underestimated. Fourth, our study did not have detailed information about death after missing events grouped by age and sex. Therefore, we could not calculate the age-standardized mortality rate and the age- and sex-specific mortality rate. Finally, this study used different time points between outcome variables and candidate explanatory variables.

In conclusion, our results showed that a high number of nursing care facilities for older adults, a high number of public health nurses, and a low proportion of people living in urban areas were associated with low missing incidence rates, and that a high proportion of people living in urban areas was associated with low death rates after dementia-related missing events. There was much prefectural difference in incidence rate and mortality rate. Men were more likely to have more missing events compared to women, and higher age was associated with higher missing events. Further individual level cohort studies should be conducted to eliminate the ecological fallacy limitation in the present study.

## APPENDIX A. SUPPLEMENTARY DATA

eFigure 1. Age-sex specific incidence rate of missing persons (a), age-standardized incidence rate (b), and result of generalized linear model (c)

eFigure 2. Scatter plot with prefectures. (a) is about the incidence rate and the number of nursing care facilities for the elderly, (b) is about the incidence rate and the number of public health nurses, (c) is about the incidence rate and the proportion of people who live in an urban area, and (d) is about the mortality rate and the proportion of people who live in an urban area.

eTable 1. Sex and age distributions of total dementia-related missing events in this study

eTable 2. Associated factors for dementia-related missing incidents in older adults
