# Supplementary material for: Incidence and Mortality of Dementia-Related Missing and Their Associated Factors: An Ecological Study in Japan
Source: J Epidemiol. 2021 Jun 5;31(6):361–8. doi: 10.2188/jea.JE20200113 (PMC8126676; doi:10.2188/jea.JE20200113)
Supplement: Supplementary file 1 [file je-31-361-s001.pdf]

eTable 1. Sex and age distributions of total dementia-related missing events in this study

	Total (n=16,927)	Men (n=9,274)	Women (n=7,653)
Age, years, n (%)			
40–49	9 (0.05)	7 (0.08)	2 (0.03)
50–59	131 (0.77)	64 (0.69)	67 (0.88)
60–69	1,353 (7.99)	707 (7.62)	646 (8.44)
70–79	6,577 (38.86)	3,431 (37.00)	3,146 (41.11)
≥80	8,857 (52.32)	5,065 (54.62)	3,792 (49.55)

eTable 2. Associated factors for dementia-related missing incidents in older adults

	Crude model		Adjusted model	
	IRR (95% CI)	*p*	IRR (95% CI)	*p*
Number of nursing care facilities for older adults	0.940 (0.910–0.970)	<0.001	0.913 (0.874–0.952)	<0.001
Number of public health nurses	0.975 (0.965–0.986)	<0.001	0.968 (0.955–0.980)	<0.001
Proportion of people who live in an urban area	1.013 (1.006–1.019)	0.001	1.021 (1.012–1.030)	<0.001

CI, confidence interval; IRR, incidence rate ratio.

Adjusted model is adjusted for the gross prefectural product.

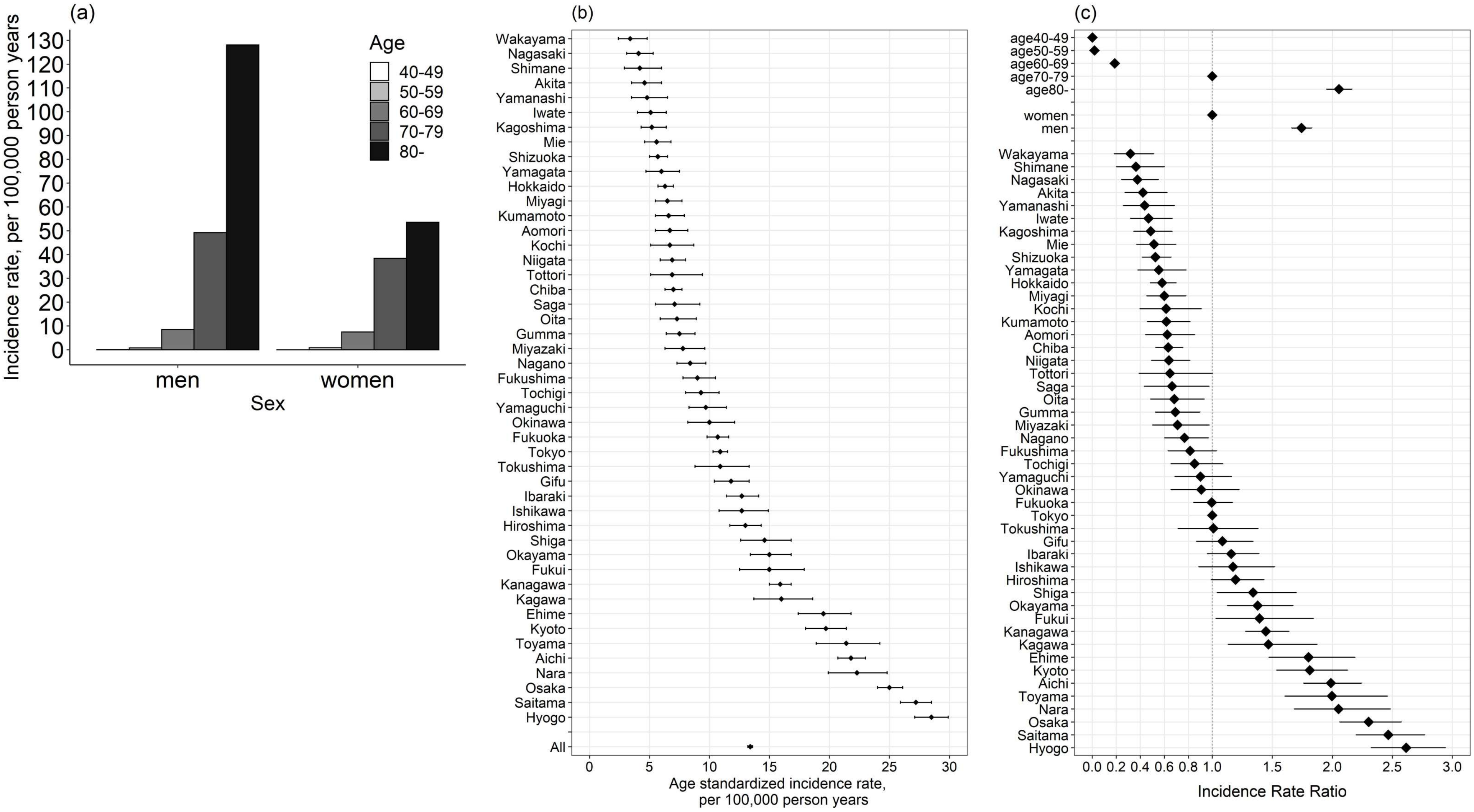

eFigure 1. Age-sex specific incidence rate of missing persons (a), age-standardized incidence rate (b), and result of generalized linear model (c)

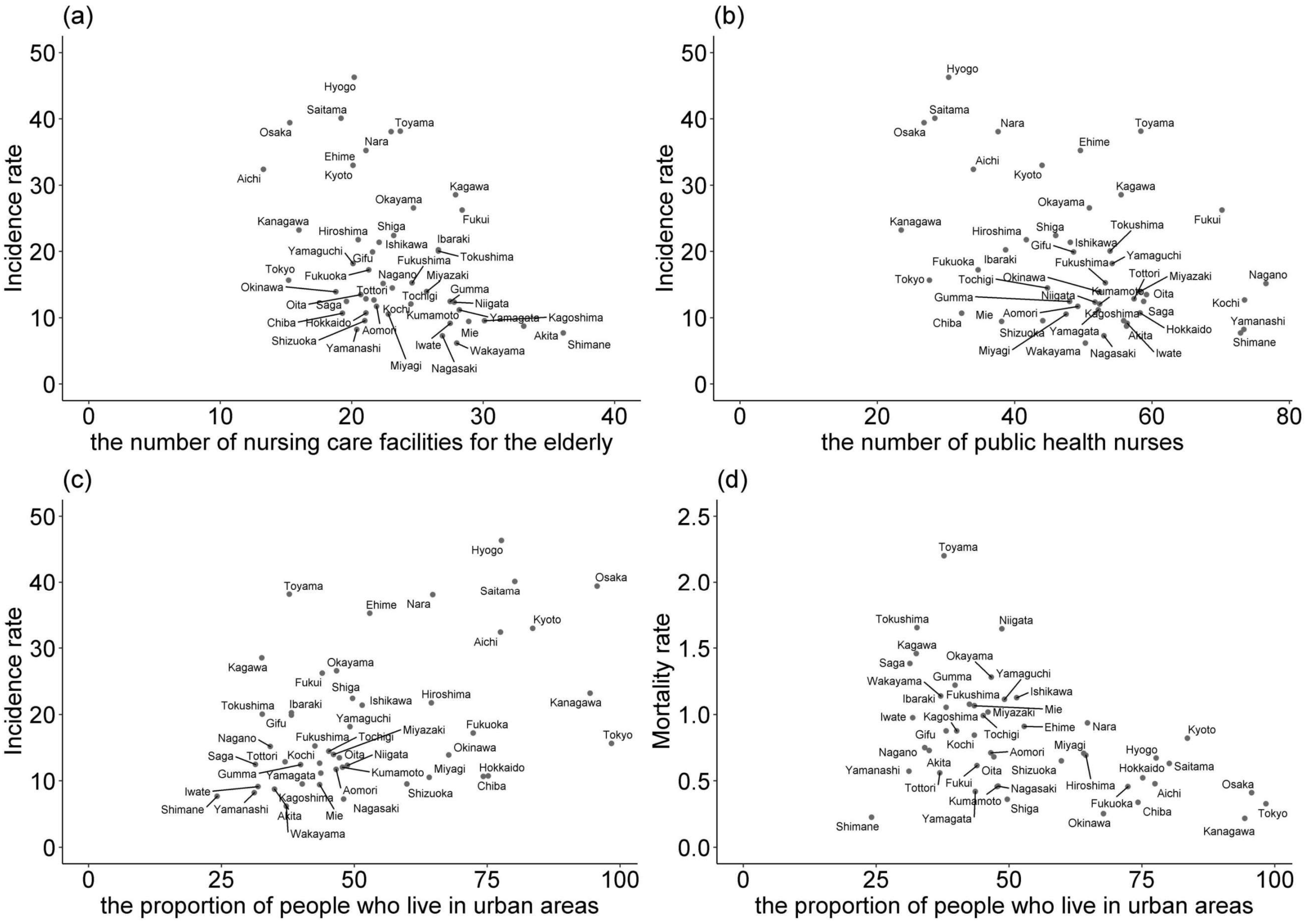

eFigure 2. Scatter plot with prefectures. (a) is about the incidence rate and the number of nursing care facilities for the elderly, (b) is about the incidence rate and the number of public health nurses, (c) is about the incidence rate and the proportion of people who live in an urban area, and (d) is about the mortality rate and the proportion of people who live in an urban area.
